# Diagnostic labelling as determinant of antibiotic prescribing for acute respiratory tract episodes in general practice

**DOI:** 10.1186/1471-2296-8-55

**Published:** 2007-09-20

**Authors:** Huug J van Duijn, Marijke M Kuyvenhoven, Hanneke M Tiebosch, François G Schellevis, Theo JM Verheij

**Affiliations:** 1 Julius Center for Health Sciences and Primary Care, University Medical Center, Location Stratenum, room 6.109, PO Box 85060, 3508 AB Utrecht, The Netherlands; 2 NIVEL, Netherlands Institute for Health Services Research, PO Box 1568, 3500 BN Utrecht, The Netherlands and VU medical centre, department of General Practice/EMGO Institute, vd Boechorststraat 7, room D539, 1081 BT Amsterdam, The Netherlands

## Abstract

**Background:**

Next to other GP characteristics, diagnostic labelling (the proportion of acute respiratory tract (RT) episodes to be labelled as infections) probably contributes to a higher volume of antibiotic prescriptions for acute RT episodes. However, it is unknown whether there is an independent association between diagnostic labelling and the volume of prescribed antibiotics, or whether diagnostic labelling is associated with the number of presented acute RT episodes and consequently with the number of antibiotics prescribed per patient per year.

**Methods:**

Data were used from the Second Dutch National Survey of General Practice (DNSGP-2) with 163 GPs from 85 Dutch practices, serving a population of 359,625 patients. Data over a 12 month period were analysed by means of multiple linear regression analysis. Main outcome measure was the volume of antibiotic prescriptions for acute RT episodes per 1,000 patients.

**Results:**

The incidence was 236.9 acute RT episodes/1,000 patients. GPs labelled about 70% of acute RT episodes as infections, and antibiotics were prescribed in 41% of all acute RT episodes. A higher incidence of acute RT episodes (beta 0.67), a stronger inclination to label episodes as infections (beta 0.24), a stronger endorsement of the need of antibiotics in case of white spots in the throat (beta 0.11) and being male (beta 0.11) were independent determinants of the prescribed volume of antibiotics for acute RT episodes, whereas diagnostic labelling was not correlated with the incidence of acute RT episodes.

**Conclusion:**

Diagnostic labelling is a relevant factor in GPs' antibiotic prescribing independent from the incidence of acute RT episodes. Therefore, quality assurance programs and postgraduate courses should emphasise to use evidence based prognostic criteria (e.g. chronic respiratory co-morbidity and old age) as an indication to prescribe antibiotics in stead of single inflammation signs or diagnostic labels.

## Background

Most antibiotics are prescribed in primary care with acute respiratory tract (RT) infections being the main indication [1]. However, there is insufficient evidence to warrant its use for most of these infections, while they are in general self-limiting [2]. Even in a low prescribing country such as the Netherlands there is an over-prescribing of antibiotics; about 50% of the antibiotic prescriptions for acute RT episodes are not in accordance with Dutch national guidelines [3-5]. Considering costs, side-effects and the growing resistance to pathogens, it is important to rationalise antibiotic prescribing as much as possible [6]. More insight into the determinants of prescribing antibiotics for acute RT episodes is therefore necessary to optimise medical education and feedback procedures [7].

Several GP characteristics have been shown to be associated with overall antibiotic prescribing rates for acute RT infections (e.g. the number of years of practice, number of patients, perceived workload, and the perception that purulent sputum is an indication for antibiotic treatment) [8-11]. Next to this, there are indications that both diagnostic labelling (i.e. the tendency to encode acute RT episodes in medical records more as infections than as symptoms) and incidence of acute RT episodes (the number of acute RT episodes per 1,000 patients presented to the GP) are associated with the volume of antibiotic prescribing [12-16].

However, it is unknown whether there is an *independent *association between diagnostic labelling and the volume of prescribed antibiotics, or whether diagnostic labelling is associated with the number of presented acute RT episodes and therefore with the volume of prescribed antibiotics. After all, labelling acute RT episodes as diagnoses instead of symptoms might be a trigger for patients to revisit the GP on a subsequent occasion, i.e. GPs who are inclined to label acute RT episodes as infections might consequently be visited more frequently by patients for these episodes.

Studies that include both diagnostic labelling and the incidence of acute RT episodes as possible determinants of antibiotic prescribing combined with GP personal characteristics are lacking. Furthermore, there are hardly any studies about possible determinants of antibiotic prescribing using data from nation-wide electronic GP data bases. Therefore the present study explores if diagnostic labelling, the incidence of acute RT episodes presented to the GP, and other GP characteristics are associated with the volume of antibiotic prescribing for acute RT episodes. This study is based on data of a nationwide study [17].

## Methods

### GPs, practices and patients

The data used in the present study were derived from the Second Dutch National Survey of General Practice (DNSGP-2), carried out by the Netherlands Institute for Health Services Research (NIVEL) in 2001 [16]. Data were used from 163 GPs from 85 practices serving a population of 359,625 patients. The patients enlisted in the participating practices were similar to the profile of the Dutch general population with respect to age, gender and type of health care insurance. There were no differences between the total population of Dutch GPs and the study group except for the type of practice: i.e. single-handed GPs where underrepresented in the study population. In all, the DNSGP-2 is assumed to provide a representative impression of the morbidity and prescribing habits in Dutch general practice.

### Morbidity and prescribing

In the DNSGP-2 study, data on morbidity and antibiotic prescribing were derived from the electronic medical records during a one-year period. Morbidity as presented to the GP was encoded using the International Classification of Primary Care version 1 (ICPC-1), [18] and contact diagnoses for the same health problem were clustered into episodes. Prescriptions were registered in a separate file using the Anatomical Therapeutic Chemical classification system (ATC) [19]. Antibiotics were identified by ATC-code J01. Acute RT episodes were identified by their ICPC codes and split up into two categories: symptoms [such as throat symptoms (R21) or cough (R05)], and infections [such as tonsillitis (R76) or pneumonia (R81)] (Table 1). We used the proportion of episodes labelled as infections as an indicator for 'diagnostic labelling', i.e. the inclination to encode acute RT episodes in medical records as infections rather than as symptoms assuming that the distribution of the various kinds of acute RT infections is about the same among the participating practices, while geographical, biological or medical factors for such a variation are not present in the Netherlands.

**Table 1:** Acute respiratory tract episodes divided into localisation and type (with the corresponding ICPC codes)

Labelled as symptom:	Labelled as infection:
	
*Upper respiratory tract:*	*Upper respiratory tract:*
	
R07 sneezing/nasal congestion	R72 strep throat
R09 sinus symptom/complaint	R74 acute upper respiratory infection
R21 throat symptom/complaint	R75 sinusitis acute/chronic
R22 tonsils symptom/complaint	R76 acute tonsillitis
H01 ear pain/earache	H71 acute otitis media/myringitis
	
*Lower respiratory tract:*	*Lower respiratory tract:*
	
R05 cough	R77 acute laryngitis/tracheitis

In 22 of the 85 practices, because the prescriptions could not be linked to a specific GP within the practice, the average practice prescription rates were allocated to all GPs of that practice.

### Questionnaire

At the beginning of the DNSGP-2 information was collected about the GPs and their practices, including age (years), gender (male/female), years since registration as a GP (years), number of enlisted patients and type of practice (single-handed yes/no). Additional information was collected by means of a written questionnaire: frequency of consulting national GP guidelines (once a week or less/more than once a week), seeing pharmaceutical representatives in the four weeks preceding completion of the questionnaire (no/yes), inclination to prescribe new drugs ranging from 1 (low) to 5 (high), views on RT symptoms and antibiotics rated on a five-point scale ranging from 1 (strongly disagree) to 5 (strongly agree) [20,21] and GPs' medical knowledge on acute RT infections and antibiotics (using a 10-question questionnaire; scored from 0 (very low) to 10 (very high)).

### Outcome measure and analysis

The outcome measure was the prescribed volume of antibiotics (the number of antibiotic prescriptions per 1,000 patients per GP per year) for acute RT episodes. To explore the association between GP characteristics and the volume of antibiotic prescribing for acute RT episodes a multiple linear regression analysis was carried out after checking for interactions and collinearity. All determinants that had a bivariate correlation with the outcome measures at p < 0.20, were included in the multiple linear regression analysis with a stepwise procedure, followed by an enter procedure. The strength of the associations between the determinants and the volume of antibiotics was described by standardised beta coefficients with 95% confidential intervals (95% CI).

All data were analysed using the Statistical Package for Social Sciences for Windows (SPSS 12.0.1).

## Results

### GPs characteristics

The GPs' mean age was 47 years and about 25% were female (Table 2). The mean period of practising since registration was 18 years and the mean number of patients was about 2,200 per GP. About 25% of all GPs had a single-handed practice, 54% of the GPs consulted national guidelines more than once a week and 56% of them had seen a pharmaceutical representative in the four weeks preceding completion of the questionnaire. The inclination to prescribe new drugs had a mean score of 2.4. In general, GPs endorsed the self-limiting character of acute RT infections (mean: 4.3), rating the seriousness of acute RT infections, the need for antibiotics in case of fever and green phlegm, and the effectiveness of antibiotics rather low (mean: 2.0, 1.7, 1.7 and 1.9 respectively). The risk of side-effects of antibiotics, the need for antibiotics in case of white spots in the throat, and the need to be consulted in case of acute RT symptoms were rated in the middle range (mean: 2.3, 2.3 and 3.5 respectively).

**Table 2:** Data on the 163 general practitioners participating in the study

Age in years (mean (SD))	47.1 (6.4)
Gender (% female)	26.4
Years since registration (mean (SD))	18.2 (8.7)
Number of patients (mean (SD))	2,197 (646)
Type of practice (% single-handed)	24.5
Consulting national guidelines for GPs (% > once a week)	54.0
Seeing pharmaceutical representatives (% yes)	56.4
Inclination to prescribe new drugs (mean (SD))*	2.4 (0.7)
Medical knowledge on respiratory tract symptoms and antibiotics (mean (SD))**	7.1 (1.5)
Views on acute respiratory tract symptoms and antibiotics (mean (SD))***	
- *Seriousness*	2.0 (0.8)
- *Self- limiting character*	4.3 (0.6)
- *Need to consult a general practitioner*	3.5 (0.8)
- *Need of antibiotics in case of fever*	1.7 (0.7)
- *Need of antibiotics in case of green phlegm*	1.7 (0.7)
- *Need of antibiotics in case of white spots in the throat*	2.3 (1.1)
- *Effectiveness of antibiotics*	1.9 (0.8)
- *Side-effects of antibiotics*	2.3 (1.0)

Acute respiratory tract episodes/1,000 patients/year (mean (SD))	236.9 (67.7)
Proportion of acute respiratory tract episodes labelled as diagnosis (mean (SD))	0.70 (0.11)
Antibiotic prescriptions for acute respiratory tract episodes per 1,000 patients/year (mean (SD))	107.4 (45.0)
Proportion of antibiotic prescriptions/acute respiratory tract episodes (mean (SD))	0.41 (0.13)

* Scale ranged as follows: 1 (low inclination) to 5 (high inclination)

** Scale ranged as follows: 1 (very low) to 10 (very high)

***Scale ranged as follows: 1 (strongly disagree) to 5 (strongly agree)

### Morbidity and prescribing

In total 236.9 acute RT episodes/1,000 patients were registered (Table 2). More than half of these episodes were for upper acute RT episodes and the remainder was for lower acute RT episodes (150.3 vs. 86.6 episodes/1,000 patients). GPs labelled 70% of all acute RT episodes as infections. In 41% of all acute RT episodes antibiotics were prescribed (97.3 antibiotic prescriptions related to 236.9 acute RT episodes), with no differences between lower and upper acute RT episodes.

### Determinants of antibiotic prescribing

Both the incidence of acute RT episodes presented to the GP (number of presented acute RT episodes/1,000 patients/year) and diagnostic labelling (proportion of acute RT episodes labelled as infections) were independently associated with the volume of antibiotic prescriptions for acute RT episodes (Table 3). There was no correlation between the incidence of acute RT episodes and diagnostic labelling (r 0.09; p = 0.24), which means that the inclination to encode acute RT episodes as infections rather than as symptoms is not correlated with the frequency of presented acute RT episodes/1,000 patients. Male gender and GPs' endorsement of the need of antibiotics in case of white spots in the throat were relatively weakly associated with the volume of antibiotic prescriptions for acute RT episodes.

**Table 3:** Correlation between GPs' characteristics and volume of antibiotic prescriptions per 1,000 patients for acute respiratory tract episodes*

**Variables in model**	**Standardised beta coefficient**
Acute respiratory tract episodes/1,000 patients/year	.67 (.57–.78)
	
Proportion of acute respiratory tract episodes labelled as infection	.24 (.13–.34)
GP's endorsement of the need of antibiotics in case of white spots in the throat	.11 (.01–.22)
Gender (male)	.11 (.00–.21)
	
*Variance explained by the model (R^2^)*	*58%*

*multiple linear regression analysis; standardised beta coefficient; 95%CI; n = 163 GPs

## Discussion

### Strengths and weaknesses of the study

The 163 participating GPs were representative for the total population of GPs in the Netherlands. The GPs' characteristics were comparable with those of the total population of Dutch GPs, except for the type of practice; single-handed GPs were under-represented in the present study. However, because this factor was not significantly correlated with the volume of antibiotics prescribed for acute RT episodes per 1,000 patients, it is unlikely that this influenced the results. In addition, the patients involved in the DNSGP-2 study reflected the general Dutch population [17].

We assume that the morbidity and antibiotic prescribing data are accurate because they were extracted from the electronic medical records of the participating practices, and the inter-observer reliability of coding episodes into the ICPC codes is high [22]. The registration covered a 12-month period for each practice, thereby eliminating seasonal influences. Considering the representativeness of the participating GPs and their patients – and the high validity of the data – the results of the present study can be assumed to validly represent morbidity and GPs prescribing behaviour in Dutch general practice.

For 56 of the 163 GPs (in 22 of the 85 practices) the volume of antibiotics prescribed was only available on the practice level. In these cases the volume of antibiotics prescribed by each GP was estimated by means of the number of prescriptions per practice. This implies a loss of variance in outcome measures so that associations in the regression analysis might have been underestimated. However, analysis of 107 GPs (163 minus 56) yielded comparable results. Finally, because our study had a cross-sectional design, we can only assume correlations and no conclusions about causal relationships can be drawn.

### Comparison with existing literature

In the present study 70% of all acute RT episodes are labelled as infections, compared with 41%–62% in other studies that labelled their acute RT episodes with a diagnosis 'assuming a bacterial infection' [13,14]. In a recent Dutch study 63% of the acute RT episodes were labelled as infections, and the antibiotic prescribing rate for all acute RT episodes was about 35% [22]. Compared with the latter study in which data were collected by hand-written prospective recording of visits we assume that our data collection from the electronic medical records is probably more accurate.

Our finding that the number of acute RT episodes presented to the GP/1,000 patients is strongly associated with the volume of prescribed antibiotics/1,000 patients for acute RT episodes has also been suggested by Ashworth et al [16]. Diagnostic labelling also turns out to be an independent determinant of the volume of antibiotics prescribed for acute RT episodes; this latter phenomenon has already been described by Howie in 1983 [12], and later confirmed by others [11,13-15]. With a correlation between the incidence of acute RT episodes and diagnostic labelling being absent we point that diagnostic labelling is directly related with the volume of antibiotic prescriptions with the number of presented acute RT episodes *not *being an intermediate factor.

Evidently, while there are no geographical, biological or medical explanations of variation in labelling acute RT episodes as infections in the Netherlands, we propose that diagnostic labelling is an arbitrary process, undoubtedly partially used to justify antibiotic prescribing. GPs may use this mechanism to defend themselves against unforeseen complications or worsening, even though these sequels will very seldom occur [12].

Signs of inflammation (such as white spots in the throat) was also found to be a determinant for over-prescribing of antibiotics in another Dutch study [5]. This supports the importance of a more selective indication setting in antibiotic prescribing. After all, as stated above, acute respiratory tract infections seldom need antibiotics, even if these infections are accompanied by green phlegm or white spots in the throat [2].

## Conclusion

The more acute RT episodes/1,000 patients are presented to a GP and the more GPs label these episodes as infections rather than as symptoms, the more GPs prescribe antibiotics for acute RT episodes, while being a male GP and endorsing the need of antibiotics in case of white spots in the throat are predictors too. This implicates that diagnostic labelling is a relevant factor in GPs' antibiotic prescribing *independent *from the incidence of acute RT episodes. Therefore, quality assurance programs and postgraduate courses should emphasise to use evidence based prognostic criteria (e.g. chronic respiratory co-morbidity and old age) as an indication to prescribe antibiotics in stead of single inflammation signs or diagnostic labels. Structured peer review groups combined with (postgraduate) education of GPs may be suitable methods to implement such recommendations.

At last, since diagnostic labelling might be influenced by e.g. reasons related to therapeutic justification, the use of diagnostic labels in research has to be handled with some caution.

## Competing interests

The author(s) declare that they have no competing interests.

## Authors' contributions

All co-authors were involved in the study design. HJD, HMT and MMK participated in the analyses. HD drafted the paper, which was revised by all co-authors. All authors read and approved the final manuscript.

## Pre-publication history

The pre-publication history for this paper can be accessed here:


